# MPK3 as a Signalling Hub in Plants: Integrating Plant Growth, Development and Stress Response

**DOI:** 10.3390/plants15060919

**Published:** 2026-03-16

**Authors:** Fan Gao, Xiushan Qi, Huihui Guo, Weijie Wang, Fengxin Liu, Xiangyue Zeng, Boyue Song, Lei Cheng, Yupeng Fan, Fanchang Zeng

**Affiliations:** 1 State Key Laboratory of Crop Biology, College of Agronomy, Shandong Agricultural University, Tai’an 271018, China; fg18325@163.com (F.G.); qixs3155@126.com (X.Q.); hhguo@sdau.edu.cn (H.G.); 13592620932@163.com (W.W.); lll666fengxin@163.com (F.L.); zxysoo@163.com (X.Z.); 18265576103@163.com (B.S.); leicheng@sdau.edu.cn (L.C.); 2 College of Life Sciences, Huaibei Normal University, Huaibei 235000, China; 3 Institute of Industrial Crops, Shandong Academy of Agricultural Sciences, Jinan 250100, China

**Keywords:** MPK3 signalling cascade, plant growth and development, stress response

## Abstract

The mitogen-activated protein kinase (MAPK) cascade constitutes a core component of signal transduction pathways in eukaryotic organisms. With its precise, efficient, and specific mechanism of action, this cascade pathway integrates, amplifies, and rapidly transmits signals. Among them, the specificity and functional diversity of the MPK3 cascade depend on the phosphorylation interaction between MKK and MPK3, as well as the specific interaction between MPK3 and its substrates. MPK3 targets an extremely diverse array of substrates, including transcription factors, RNA-binding proteins, enzymes, and transporters. The summary of the regulatory role of the MPK3 signal mainly focuses on three functional mechanisms: The most well-known regulatory mechanism is to recognize and phosphorylate substrate proteins or transcription factors, thereby affecting the stability and transcriptional activity of downstream substrates, and thus regulating the transcriptional regulatory activity and expression of downstream genes. MPK3 can also participate in downstream functional regulation by triggering the MAPKKK-MKK4/5-MPK3/6 signaling pathways or feedback mechanisms. MPK3 can exert regulatory effects independently or together with MPK6. The redundancy of the MPK3/6 function is related to the synergistic effect of the component cascade reaction, as well as the dose-dependent activation effect. This article presents a comprehensive synthesis of the latest research progress on the regulatory role of MPK3, in plant growth, development, and stress adaptation and defence. Moreover, it provides critical evaluations and forward-looking perspectives on the future investigation of the underlying molecular mechanisms governing MPK3-mediated regulation.

## 1. Introduction

As an evolutionarily conserved signalling module, the mitogen-activated protein kinase (MAPK) cascade mediates environmental adaptation and developmental events in all eukaryotes, serving as a central hub in diverse physiological and developmental pathways across plants, animals, and humans [1,2]. In plant MAPK research, the MAPK family in *Arabidopsis thaliana* is the most extensively characterized group. Recent investigations have demonstrated that the genome of this organism encodes 80 MAPKKKs, 10 MAPKKs, and 20 MAPKs, with the classification and mutual recognition of these family members primarily based on their functional features and evolutionary relationships [3]. Among them, MPK3 serves as a signalling hub, and the mechanisms of its specificity and functional diversity still hold significant research value and pose many unresolved problems. The central question is how a single MAP kinase achieves such regulatory breadth. Recent years have witnessed significant advances in plant MPK3 research, with numerous studies demonstrating the involvement of MPK3 cascades in regulating embryonic development, stomatal development, cell division, differentiation, and programmed cell death [1,3,4,5,6,7]. Furthermore, MPK3 signalling modulates plant responses to various stresses, including pathogen infection, drought, salinity, low temperature, and osmotic stress [8,9,10,11,12,13]. MPK3 can function independently or in conjunction with MPK6 to exert regulatory effects. They function redundantly in innate immunity [14] and abiotic stress adaptation [15]. These kinases also exhibit pleiotropic effects in various reactive oxygen species (ROS)-controlled processes and ozone stress tolerance [15,16,17]. Their multifunctionality and signalling specificity appear to arise from their ability to phosphorylate distinct substrates. In certain contexts, these two highly homologous proteins exhibit functional overlap [18].

In conclusion, MPK3 influences the expression of related genes by integrating hormone signals, environmental signals, and intracellular endogenous signals as a signalling hub in plants. It serves as an important regulatory component for plant growth and development, as well as their adaptation to the complex and dynamically changing natural environment.

## 2. MPK3 Signalling Cascade

The MPK3 cascade forms a three-tiered kinase signalling relay, in which MAPKKK undergoes phosphorylation and subsequent activation in response to external and endogenous stimuli [19]. The activated MAPKKK subsequently catalyzes the phosphorylation of the downstream MKK4/5, and the latter sequentially triggers the phosphorylation of MPK3 [20]. Through its precise, efficient, and specific mode of action, the MPK3 cascade enables signal integration, amplification, and rapid transduction [9] MPK3 acts as a key signal transducer, transmitting signals from the cell surface to the nuclear compartment while catalyzing protein phosphorylation reactions. The specific signal transduction of the MPK3 cascade depends on the phosphorylation interaction between MKK and MPK3, as well as the specific protein substrates phosphorylated by MPK3 [21]. In terms of molecular function, activated MPK3 can phosphorylate and activate various substrate proteins in the cytoplasm and nucleus, significantly influencing the subcellular localization, activity state, stability, and transcriptional regulatory functions of these substrate proteins [22]. MPK3 targets an extremely diverse array of substrates, including transcription factors, RNA-binding proteins, enzymes, and transporters. This suggests that the phosphorylation-mediated regulatory functions of MPK3 are highly versatile [23]. The phosphorylation of these target proteins plays a central role in both biotic and abiotic stress responses [24]. The summary of the regulatory role of the MPK3 signal mainly focuses on three functional mechanisms: The most well-known regulatory mechanism is to recognize and phosphorylate substrate proteins or transcription factors, thereby affecting the stability and transcriptional activity of downstream substrates, and thus regulating the transcriptional regulatory activity and expression of downstream genes. MPK3 can also participate in downstream functional regulation by triggering the MAPKKK-MKK4/5-MPK3/6 signaling pathways or feedback mechanisms.

## 3. MPK3 and MPK6: Redundancy vs. Divergence

These two highly homologous proteins exhibit largely overlapping functions and substrate preferences. Both MPK3 and MPK6 are activated by the upstream MAPKs MKK4 and MKK5 [14]. In contrast to MPK6, MPK3 is regulated not only at the post-translational level but also via additional regulatory mechanisms. Accordingly, the transcript and protein abundance of MPK3 accumulate in a stress-dependent manner [10]. The redundancy of the MPK3/6 function is related to the synergistic effect of the component cascade reaction, as well as the dose-dependent activation effect. Furthermore, in most cases, MPK3 and MPK6 act together. The sole functioning of MPK3 alone and the functional redundancy of MPK3 and MPK6 occur only in a few instances. We will illustrate this with specific examples later in the text.

## 4. MPK3 in Plant Growth and Developmental Patterning

Plant cell growth and developmental patterning are intricately interrelated. We provide a comprehensive review from three aspects: meristem maintenance and cell cycle control, asymmetric division and tissue morphogenesis, and reproductive and specialized developmental processes. MPK3 phosphorylates downstream substrate proteins or transcription factors, and by dynamically activating the MAPKKK-MKK4/5-MPK3/6 signaling pathway, it functions as a conserved mechanism. Elucidating the regulatory mechanisms underlying the mutual promotion and antagonism between plant growth and developmental patterning, as well as the pivotal role of the MPK3 cascade as a molecular bridge and mediator in this regulatory network, will also serve as key research avenues for investigating the underlying molecular mechanisms in the future.

(1) **Meristem maintenance and cell cycle control**

During the organ developmental process of multicellular organisms, cell proliferation and differentiation are orchestrated in a spatiotemporally coordinated manner [25]. This process involves a wide range of molecular pathways, among which MPK3 plays a pivotal role. The exogenous CLV3p signal triggers rapid signal transduction in the shoot apical meristem through the dynamic activation of MPK3 and MPK6, potentially enhancing WUS activity by stimulating the CLV3-CLV1/BAM1 signalling pathway, which redundantly affects the expression of marker genes related to shoot apical meristem development and control the homeostasis of stem cells in the shoot apical meristem [1]. Ref. [26] identified that the YDA-MKK4/MKK5-MPK3/MPK6 signalling cascade plays a critical role in root meristem development by maintaining mitotic activity in the root apical meristem, thereby regulating Arabidopsis stem cell populations and primary root growth. MPK3 can also function independently in certain circumstances. According to reports, MPK3 exhibits synergistic interactions with the cyclin-dependent kinase inhibitor protein KRP3 at the S-phase checkpoint. KRP3 regulates protein stability through MPK3-mediated phosphorylation, thereby coordinating the balance between cell division and elongation, thereby influencing the plant type in rice [27].

(2) **Asymmetric division and tissue morphogenesis**

MPK3 and MPK6 are both activated by auxin, and have their encoding genes highly expressed in the hypocotyl. The MPK3/MPK6 protein kinase phosphorylates and mediates the stability of the GRF4 protein, which promotes the nuclear accumulation of BZR1 protein and subsequently facilitates the elongation of lower hypocotyl meristem cells through a specific molecular mechanism [28]. Additionally, research findings have demonstrated that GhWRKY16 undergoes phosphorylation by GhMPK3-1 that augments its capacity to trigger the transcriptional activation of downstream fibermeristem-associated genes, consequently accelerating the elongation of cotton fibers [5]. MPK3/6 can also participate in downstream functional regulation by triggering the MAPKKK-MKK4/5-MPK3/6 signaling pathways: The MKK4/MKK5-MPK3/MPK6 signalling module exerts a regulatory function in the development and morphogenesis of stomata downstream of YDA, and acts as an essential regulatory component for orchestrating cell fate determination during stomatal specification in epidermal and pavement cells [29]. MPK3/6 regulate stomatal asymmetric division via phosphorylation of the SPCH transcription factor and reinforce the BASL-MAPK signalling cascade by phosphorylating BASL [30]. Ref. [31] showed that the MAPKKK-MKK4/5-MPK3/6 signalling cascade has dual functions in mesophyll and epidermal cells, regulating the expression of STOMAGEN (STO) peptide ligands to coordinate stomatal development. MPK3/6 is a critical factor for lateral root emergence through the epidermis. The IDA-HAE/HSL2 signalling pathway triggers the MKK4/MKK5-MPK3/MPK6 phosphorylation cascade, which in turn fine-tunes the transcriptional profile of genes associated with cell wall remodeling in lateral root primordium (LRP) cells. This regulatory mechanism facilitates pectin degradation within the middle lamella, enhances intercellular separation, and thereby enables the protrusion of lateral root primordia through the epidermal layer, a pivotal step for lateral root morphogenesis [32].

(3) **Reproductive and specialized developmental processes**

During plant embryogenesis, Arabidopsis MPK3 and MPK6 phosphorylate WRKY2, thereby enhancing the transcriptional activity of its downstream target gene WOX8, which is essential for zygote division and suspensor formation, thereby influencing embryonic development patterns [33,34,35,36]. It has been reported that Arabidopsis WRKY34 is phosphorylated by MPK3 and MPK6, which maintains pollen viability and promotes pollen tube growth. The MAPK-WRKY signalling module represents a novel signalling pathway specific to particular stages of pollen development, functioning in the early stages and being crucial for its internal processes [37]. MPK3/6 exerts a pivotal regulatory role in modulating the stability of Aux/IAA proteins within the auxin signalling pathway via phosphorylation-mediated post-translational modifications. In vitro experimental results have confirmed that the IAA8, IAA3 and IAA7 proteins serve as phosphorylation substrates for both MPK3 and MPK6 kinases, significantly enhancing their stability and thereby regulating the development of the main root in plants [38]. This process influences the expression of auxin-responsive genes and maintains basal auxin signal transduction during plant stress adaptation [39]. Moreover, the MPK3/MPK6 cascade exerts a spatiotemporally constrained regulatory role throughout plant ontogeny by triggering the MAPKKK-MKK4/5-MPK3/6 signaling pathways: The YDA-MKK4/MKK5-MPK3/MPK6 cascade reaction functions downstream of the ER receptor. It modulates pedicel elongation and inflorescence architecture patterning, and thus serves as a key determinant of the morphological characteristics of plant reproductive organs [6]. Ref. [40] found that high concentrations of auxin at the side root initiation site are recognized by the transmembrane kinases TMK1 and TMK4. This recognition activates downstream MPK3/6 to form signaling pathways either by directly phosphorylating MKK4/5 or by enhancing the phosphorylation of MPKKK on MKK4/5, thus regulating the direction of cell division during side root development and ultimately facilitating the regulation of side root formation. MPK3 can also function redundantly alongside MPK6 in certain circumstances. Ref. [35] provided experimental evidence that there is a certain degree of functional redundancy between MPK3 and MPK6: in the context of MPK6 mutants, Arabidopsis exhibits abnormal ovary development, with cell division stalling at the later stage, resulting in the loss of female reproductive function. However, when MPK3 is absent, due to the dependent activation effect of the gene dosage of MPK3 and MPK6, the activation effect of MPK6 will be enhanced, and the plant will be fertile. These kinases mediate the proliferation dynamics of epidermal cells during the late developmental stage of ovules.

Currently, research into the regulatory roles of MPK3 in plant cell growth and developmental patterning has been most extensively conducted on stomatal asymmetric division and lateral root development. In contrast, relevant investigations into plant morphogenesis and somatic cell regeneration remain scarce, which may emerge as a novel research focus and key breakthrough point for mechanistic studies in this field going forward.

## 5. MPK3 in Stress Adaptation and Defence

To adapt to diverse environmental stresses, plants have evolved a variety of stress resistance mechanisms. The MPK3 cascade and phytohormone signal transduction form an intricate and elaborate regulatory network, which readily generates feedback and dual regulatory loops to enable plastic adaptation to the dynamic and complex changes in the external environment. This regulatory system can thus serve as a valuable reference model for the identification of novel phytohormone-responsive genes and their interaction mechanisms in future research. The regulation of transcription factors and target genes by MPK3-mediated phosphorylation not only represents the core content of future research but also serves as the molecular basis for constructing intricate regulatory networks.

(1) **Abiotic stress regulation**

Ref. [13] have demonstrated that in *Arabidopsis thaliana*, MPK3 and MPK6 act as essential protein kinases implicated in the cold stress signalling pathway. These kinases phosphorylate ICE1 protein and impair its stability and transcriptional activity; this consequently represses CBF gene expression and exerts a negative regulatory effect on the cold tolerance of plants. Specifically, ref. [41] revealed an antagonistic biochemical mechanism: under hypoxic conditions, MPK3/MPK6 interact with and phosphorylate the transcription factor STOP1, and this process competes with PUB24-mediated ubiquitin-dependent protein degradation. This antagonism regulates the nuclear accumulation of STOP1 and activates the transcription of glutamate dehydrogenase 1 (GDH1) and GDH2, thereby maintaining cellular homeostasis during hypoxia and further modulating the hypoxic response in *Arabidopsis thaliana*. Ref. [42] discovered that under salt stress conditions, MPK3 and MPK6 phosphorylate the ARR1/10/12 proteins, accelerating their degradation and negatively regulating their stability, ultimately enhancing the plant’s tolerance to salt stress. Ref. [43] demonstrated that GhPIPL7 activates the MPK3/6 signaling cascade, thereby promoting the transcription and expression of downstream target genes to mediate plant responses to salt stress-induced signaling pathways. Furthermore, MPK3 can also participate in downstream functional regulation by forming a feedback regulatory mechanism. Ref. [44] reported that MPK3 and MPK6 bind to phosphatidic acid (PA) in vitro, promoting their interaction with and phosphorylation of AP2.12, a key transcription factor in hypoxia signalling, which modulates its activity in vivo. Furthermore, MPK3/MPK6 form a negative feedback signalling mechanism with phospholipase D(PLD) isoforms PLDa1/PLDd to regulate PA production. PA modulates Arabidopsis submergence tolerance by MPK3/6-mediated hypoxia signalling. Ref. [45] demonstrated that MPK3 interacts with and phosphorylates the VIP1 protein, and its activity is regulated by the upstream negative regulator AGB1. This forms an AGB1-MPK3-VIP1 negative feedback regulatory mechanism, which mediates plant responses to the perception and transduction of abscisic acid (ABA) signals. It is the case where MPK3 functions independently under specific circumstances.

(2) **Biotic stress and immunity**

Ref. [46] found that the MAPK signaling module is crucial for soybean to establish resistance against Soybean cyst nematode (SCN) infection. Specifically, GmMPK3 and GmMPK6 phosphorylate GmCDL1 to prevent its proteasome-mediated degradation, thereby regulating immune responses in soybean and enhancing SCN resistance. Additional studies indicate that MPK3/MPK6 promote pathogen-induced ethylene biosynthesis through phosphorylation of ACS2 and ACS6 substrates, thereby stabilizing the ACS2 and ACS6 proteins [47,48,49,50]. Beyond elevating the protein abundance of ACS2 and ACS6 through post-translational modifications, MPK3 and MPK6 also mediate the phosphorylation and activation of the transcription factor WRKY33, thereby establishing a positive feedback regulatory loop to counteract *Pseudomonas syringae* infection [51]. Furthermore, MPK3 and MPK6 play redundant regulatory roles in specific contexts of plant-pathogen interactions: Under PAMP treatment, MYB44 binds to the promoters of MPK3 and MPK6 to activate their expression, which in turn leads to the phosphorylation of MPK3 and MPK6 proteins. Through transcriptional and post-transcriptional regulation, MYB44 works in concert with MPK3 and MPK6 to form a functional cascade reaction. Phosphorylated MPK3 and MPK6 then phosphorylate MYB44 in a functionally redundant manner, further activating downstream defense responses and enhancing resistance to *Pseudomonas syringae* [52].

(3) **Dual regulatory roles and context dependence**

MPK3 and MPK6 phosphorylate the ERF1A protein, mediating the negative feedback regulation of ethylene biosynthesis induced by *Pseudomonas syringae*, while also upregulating the expression of defense genes, and promote the resistance of *Arabidopsis thaliana* to *Pseudomonas syringae*. This dual regulatory mechanism enhances the resistance of *Arabidopsis thaliana* against *Pseudomonas syringae*, thereby strengthening its capacity to defend against pathogenic infection [53]. The abscisic acid (ABA) signalling network features MPK3 as a key component that exhibits the unique characteristic of a dual-action mechanism. In *Arabidopsis thaliana*, abscisic acid (ABA) is capable of directly triggering the MPK3/MPK6 cascade signalling pathway, wherein the activated MPK3 mediates the phosphorylation of ABI5—a core transcription factor. Additionally, direct binding of ABI5 to the promoter sequence of MPK3 enables the regulation of its transcriptional expression, forming an ‘MPK3-ABI5’ feedback loop that achieves dynamic and precise regulation of ABA signal intensity [54].

## 6. Conclusions and Perspectives

**Figure 1:**
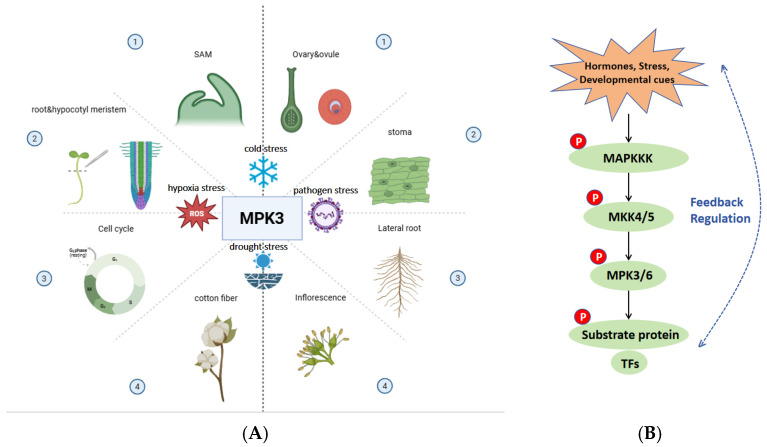
Summary diagram of MPK3’s versatile role in regulating plant growth, development and stress response. (**A**) MPK3 plays a crucial role in regulating the proliferation and differentiation of plant shoot apical meristems, cell cycle progression, hypocotyl meristem cell elongation, cotton fiber elongation, inflorescence morphogenesis, the development of stomata, lateral roots, ovaries and ovules, as well as regulating responses to drought, cold, hypoxia, and pathogen stresses. (**B**) MPK3 signal cascade diagram. Affected by the activation of upstream factors, the activated MAPKKK subsequently catalyzes the phosphorylation of the downstream MKK4/5, and the latter sequentially triggers the phosphorylation of MPK3/6, then MPK3/6 phosphorylates its specific substrate protein or transcription factor. The feedback regulation exists in certain situations between the upstream factors and downstream substrates. All icons are from BioRender.com.

As core signalling molecules, MAPKs act downstream of sensors or receptors, coordinating various cellular responses in plants [55]. The MPK3 signalling cascade constitutes a complex, mutually interconnected network that operates intracellularly [56]. MPK3 serves as an indispensable regulatory component for plant growth and development, with a pivotal role in adapting to the complex and dynamically changing natural environment (Figure 1). The central question is how a single MAP kinase achieves such regulatory breadth. MPK3 regulates downstream gene transcriptional regulatory activity and expression by recognizing and phosphorylating substrate proteins or transcription factors, thereby influencing the stability and transcriptional activity of downstream substrates. MPK3 can also participate in downstream functional regulation by triggering the MAPKKK-MKK4/5-MPK3/6 signaling pathways or feedback mechanisms. In addition, in most cases, MPK3 and MPK6 act together. The sole functioning of MPK3 alone and the functional redundancy of MPK3 and MPK6 occur only in a few instances. The redundancy of the MPK3/6 function is related to the synergistic effect of the component cascade reaction, as well as the dose-dependent activation effect. Nevertheless, several unaddressed issues pertaining to MPK3’s functional network persist: To begin with, the regulatory mechanism underlying MPK3’s substrate specificity and the dynamic imaging of MPK3 activation under combined stress conditions in distinct developmental phases remains elusive. We need to base on structural studies on substrate docking interfaces. In addition, the comparative phosphoproteomics between mpk3 and mpk6 mutants—especially their cooperative or antagonistic interactions within intricate signalling networks—remain to be further elucidated via technologies such as single-cell sequencing. Thirdly, the conservation and specificity patterns of MPK3 functions across different species have not been systematically summarized, which limits its cross-species application in crop breeding. Going forward, the combination of multi-omics technologies and cross-species research is expected to facilitate an in-depth and holistic analysis of the MPK3 regulatory network, providing innovative strategies for the targeted modulation of plant growth and developmental processes.

## Data Availability

No new data was generated during the study.

## References

[1] Lee H., Jun Y.S., Cha O.K., Sheen J. (2019). Mitogen-activated protein kinases MPK3 and MPK6 are required for stem cell maintenance in the Arabidopsis shoot apical meristem. Plant Cell Rep..

[2] Somssich M., Je B.I., Simon R., Jackson D. (2016). CLAVATA-WUSCHEL signalling in the shoot meristem. Development.

[3] Group M. (2002). Mitogen-activated protein kinase cascades in plants: A new nomenclature. Trends Plant Sci..

[4] Jonak C., Okresz L., Bogre Hirt H. (2002). Complexity, cross talk and integration of plant MAP kinase signalling. Curr. Opin. Plant Biol..

[5] Wang N.N., Li Y., Chen Y.H., Lu R., Zhou L., Wang Y., Zheng Y., Li X.B. (2021). Phosphorylation of WRKY16 by MPK3-1 is essential for its transcriptional activity during fiber initiation and elongation in cotton (*Gossypium hirsutum*). Plant Cell.

[6] Meng X., Wang H., He Y., Liu Y., Walker J.C., Torii K.U., Zhang S. (2012). A MAPK cascade downstream of ERECTA receptor-like protein kinase regulates Arabidopsis inflorescence architecture by promoting localized cell proliferation. Plant Cell.

[7] Cho S.K., Larue C.T., Chevalier D., Wang H., Jinn T.L., Zhang S., Walker J.C. (2008). Regulation of floral organ abscission in *Arabidopsis thaliana*. Proc. Natl. Acad. Sci. USA.

[8] de Zelicourt A., Colcombet J., Hirt H. (2016). The role of MAPK modules and ABA during abiotic stress signalling. Trends Plant Sci..

[9] Danquah A., de Zelicourt A., Colcombet J., Hirt H. (2014). The role of ABA and MAPK signalling pathways in plant abiotic stress responses. Biotechnol. Adv..

[10] Nakagami H., Pitzschke A., Hirt H. (2005). Emerging MAP kinase pathways in plant stress signalling. Trends Plant Sci..

[11] Zhang S., Klessig D.F. (2001). MAPK cascades in plant defense signalling. Trends Plant Sci..

[12] Rodriguez M.C.S., Petersen M., Mundy J. (2010). Mitogen-activated protein kinase signalling in plants. Annu. Rev. Plant Biol..

[13] Li H., Ding Y., Shi Y., Zhang X., Zhang S., Gong Z., Yang S. (2017). MPK3- and MPK6-mediated ICE1 phosphorylation negatively regulates ICE1 stability and freezing tolerance in Arabidopsis. Dev. Cell..

[14] Asai T., Tena G., Plotnikova J., Willmann M.R., Chiu W.L., Gómez-Gómez L., Boller T., Ausubel F.M., Sheen J. (2002). MAP kinase signalling cascade in Arabidopsis innate immunity. Nature.

[15] Miles G.P., Samuel M.A., Zhang Y., Ellis B.E. (2005). RNA interference-based (RNAi) suppression of AtMPK6, an Arabidopsis mitogen-activated protein kinase, results in hypersensitivity to ozone and misregulation of AtMPK3. Environ. Pollut..

[16] Bergmann D.C., Lukowitz W., Somerville C.R. (2004). Stomatal development and pattern controlled by a MAPKK kinase. Science.

[17] Wang H., Chevalier D., Larue C., Ki Cho S., Walker J.C. (2007). The protein phosphatases and protein kinases of *Arabidopsis thaliana*. Arab. Book.

[18] Pitzschke A., Hirt H. (2009). Disentangling the complexity of mitogen-activated protein kinases and reactive oxygen species signalling. Plant Physiol..

[19] Sinha A.K., Jaggi M., Raghuram B., Tuteja N. (2011). Mitogen-activated protein kinase signalling in plants under abiotic stress. Plant Signal Behav..

[20] Bigeard J., Hirt H. (2018). Nuclear signalling of plant MAPKs. Front. Plant Sci..

[21] Dóczi R., Bögre L. (2018). The Quest for MAP Kinase Substrates: Gaining Momentum. Trends Plant Sci..

[22] Whitmarsh A.J. (2007). Regulation of gene transcription by mitogen-activated protein kinase signalling pathways. Biochim. Biophys. Acta.

[23] Pitzschke A. (2015). Modes of MAPK substrate recognition and control. Trends Plant Sci..

[24] Bhagat P.K., Verma D., Singh K., Badmi R., Sharma D., Sinha A.K. (2022). Dynamic phosphorylation of miRNA biogenesis factor HYL1 by MPK3 involving nuclear-cytoplasmic shuttling and protein stability in Arabidopsis. Int. J. Mol. Sci..

[25] Araki S., Ito M., Soyano T., Nishihama R., Machida Y. (2004). Mitotic cyclins stimulate the activity of c-Myb-like factors for transactivation of G2/M phase-specific genes in tobacco. J. Biol. Chem..

[26] Shao Y., Yu X., Xu X., Li Y., Yuan W., Xu Y., Mao C., Zhang S., Xu J. (2020). The YDA-MKK4/MKK5-MPK3/MPK6 cascade functions downstream of the RGF1-RGI ligand-receptor pair in regulating mitotic activity in root apical meristem. Mol. Plant..

[27] Banerjee G., Jonwal S., Rengasamy B., Uttam P., Dhanraj S., Mohit M., Alok K.S. (2025). KRP3 stability controls rice plant architecture and productivity via MPK3-mediated phosphorylation. Plant Biotechnol. J..

[28] Yu Z., Ma J., Zhang M., Li X., Sun Y., Zhang M., Ding Z. (2023). Auxin promotes hypocotyl elongation by enhancing BZR1 nuclear accumulation in Arabidopsis. Sci. Adv..

[29] Wang H., Ngwenyama N., Liu Y., Walker J.C., Zhang S. (2007). Stomatal development and patterning are regulated by environmentally responsive mitogen-activated protein kinases in Arabidopsis. Plant Cell.

[30] Zhang Y., Wang P., Shao W., Zhu J.K., Dong J. (2015). The BASL polarity protein controls a MAPK signalling feedback loop in asymmetric cell division. Dev. Cell.

[31] Wu M., Wang S., Ma P., Li B., Hu H., Wang Z., Qiu Q., Qiao Y., Niu D., Lukowitz W. (2024). Dual roles of the MPK3 and MPK6 mitogen-activated protein kinases in regulating Arabidopsis stomatal development. Plant Cell.

[32] Zhu Q., Shao Y., Ge S., Zhang M., Zhang T., Hu X., Liu Y., Walker J., Zhang S., Xu J. (2019). A MAPK cascade downstream of IDA-HAE/HSL2 ligand-receptor pair in lateralroot emergence. Nat. Plants.

[33] Ueda M., Aichinger E., Gong W., Groot E., Verstraeten I., Vu L.D., De Smet I., Higashiyama T., Umeda M., Laux T. (2017). Transcriptional integration of paternal and maternal factors in the Arabidopsis zygote. Genes Dev..

[34] Lukowitz W., Roeder A., Parmenter D., Somerville C. (2004). A MAPKK kinase gene regulates extra-embryonic cell fate in Arabidopsis. Cell.

[35] Wang H., Liu Y., Bruffett K., Lee J., Hause G., Walker J.C., Zhang S. (2008). Haplo-insufficiency of MPK3 in MPK6 mutant background uncovers a novel function of these two MAPKs in Arabidopsis ovule development. Plant Cell.

[36] Bayer M., Nawy T., Giglione C., Galli M., Meinnel T., Lukowitz W. (2009). Paternal control of embryonic patterning in Arabidopsis thaliana. Science.

[37] Guan Y., Meng X., Khanna R., LaMontagne E., Liu Y., Zhang S. (2014). Phosphorylation of a WRKY transcription factor by MAPKs is required for pollen development and function in Arabidopsis. PLoS Genet..

[38] Noryang S., Manna M., Verma N., Singh K., Tayyeba S., Sinha A.K. (2025). Mitogen-activated protein kinase 3/6 regulates the stability of AtIAA3 and AtIAA7 during auxin signalling in Arabidopsis. Plant Sci..

[39] Wang C., Li X., Zhao H., Cui X., Xu W., Li K., Xu Y., Yu Z., Yu L., Guo R. (2025). Mitogen-activated protein kinases 3/6 reduce auxin signalling via stabilizing indoleacetic acid-induced proteins 8/9 in plant abiotic stress adaptation. Int. J. Mol. Sci..

[40] Huang R., Zheng R., He J., Zhou Z., Wang J., Xiong Y., Xu T. (2019). Noncanonical auxin signalling regulates cell division pattern during lateral root development. Proc. Natl. Acad. Sci. USA.

[41] Wang J.H., Zhou Y., Su G.Z., Song Q.Q., Lin G.F., Xing Y., Chen Q.F., Yu L.J., Su S.H., Xie R.H. (2025). MPK3- and MPK6-mediated phosphorylation of STOP1 triggers its nuclear stabilization to modulate hypoxia responses in Arabidopsis. Plant Cell.

[42] Yan Z., Wang J., Wang F., Xie C., Lv B., Yu Z., Dai S., Liu X., Xia G., Tian H. (2021). MPK3/6-induced degradation of ARR1/10/12 promotes salt tolerance in Arabidopsis. EMBO Rep..

[43] Liang H.C., Wang T., Yang P.Y., He S.C., Chang S.W., Lu S.Q., Xiao F. (2025). A small peptide enhances cotton salt tolerance via MPK3/6 activation: Functional discovery of GhPIPL7 in salt stress signaling. Ind. Crops Prod..

[44] Zhou Y., Zhou D.M., Yu W.W., Shi L.L., Zhang Y., Lai Y.X., Huang L.P., Qi H., Chen Q.F., Yao N. (2022). Phosphatidic acid modulates MPK3-and MPK6-mediated hypoxia signalling in Arabidopsis. Plant Cell.

[45] Xu D., Tang W., Ma Y., Wang X., Yang Y., Wang X., Xie L., Huang S., Qin T., Tang W. (2024). Arabidopsis G-protein β subunit AGB1 represses abscisic acid signaling via attenuation of the MPK3-VIP1 phosphorylation cascade. J. Exp. Bot..

[46] Zhang L., Zhu Q., Tan Y.H., Deng M.M., Zhang L., Cao Y.R., Guo X.L. (2024). Mitogen-activated protein kinases MPK3 and MPK6 phosphorylate receptor-like cytoplasmic kinase CDL1 to regulate soybean basal immunity. Plant Cell.

[47] Li G., Meng X., Wang R., Mao G., Han L., Liu Y., Zhang S. (2012). Dual-level regulation of ACC synthase activity by MPK3/MPK6 cascade and its downstream WRKY transcription factor during ethylene induction in Arabidopsis. PLoS Genet..

[48] Joo S., Liu Y., Lueth A., Zhang S. (2008). MAPK phosphorylation-induced stabilization of ACS6 protein is mediated by the non-catalytic C-terminal domain, which also contains the cis-determinant for rapid degradation by the 26S proteasome pathway. Plant J..

[49] Han L., Li G.J., Yang K.Y., Mao G., Wang R., Liu Y., Zhang S. (2010). Mitogenactivated protein kinase 3 and 6 regulate Botrytis cinerea-induced ethylene production in Arabidopsis. Plant J..

[50] Xu J., Zhang S. (2014). Regulation of ethylene biosynthesis and signalling by protein kinases and phosphatases. Mol. Plant.

[51] Mao G., Meng X., Liu Y., Zheng Z., Chen Z., Zhang S. (2011). Phosphorylation of a WRKY transcription factor by two pathogen-responsive MAPKs drives phytoalexin biosynthesis in Arabidopsis. Plant Cell.

[52] Wang Z., Li X., Yao X., Ma J., Lu K., An Y., Sun Z., Wang Q., Zhou M., Qin L. (2023). MYB44 regulates PTI by promoting the expression of EIN2 and MPK3/6 in Arabidopsis. Plant Commun..

[53] Wang X., Meng H., Tang Y., Zhang Y., He Y., Zhou J., Meng X. (2022). Phosphorylation of an ethylene response factor by MPK3/MPK6 mediates negative feedback regulation of pathogen-induced ethylene biosynthesis in Arabidopsis. J. Genet. Genom..

[54] Bhagat P.K., Verma N., Pandey S., Verma D., Sinha A.K. (2025). MPK3 mediated phosphorylation inhibits the dimerization of ABI5 to fine-tune the ABA signalling in Arabidopsis. Plant Physiol. Biochem..

[55] Zhang M.M., Zhang S.Q. (2022). Mitogen-activated protein kinase cascades in plant signalling. J. Integr. Plant Biol..

[56] Pedley K.F., Martin G.B. (2005). Role of mitogen-activated protein kinases in plant immunity. Curr. Opin. Plant Biol..

